# Association of Polymorphisms in Genes Involved in One-Carbon Metabolism with *MTHFR* Methylation Levels

**DOI:** 10.3390/ijms20153754

**Published:** 2019-07-31

**Authors:** Fabio Coppedè, Andrea Stoccoro, Pierpaola Tannorella, Roberta Gallo, Vanessa Nicolì, Lucia Migliore

**Affiliations:** 1 Department of Translational Research and of New Surgical and Medical Technologies, University of Pisa, Via Roma 55, 56126 Pisa, Italy; 2 Unit of Genetics of Neurodegenerative and Metabolic Diseases, Fondazione IRCCS Istituto Neurologico Carlo Besta, 20133 Milan, Italy; 3 Doctoral School in Genetics, Oncology and Clinical Medicine, Department of Medical Biotechnologies, University of Siena, 53100 Siena, Italy

**Keywords:** methylenetetrahydrofolate reductase, MTHFR, methylation, one-carbon metabolism, folate metabolism, polymorphisms, DNMT3B, epigenetics

## Abstract

Methylenetetrahydrofolate reductase (MTHFR) is a pivotal enzyme in the one-carbon metabolism, a metabolic pathway required for DNA synthesis and methylation reactions. *MTHFR* hypermethylation, resulting in reduced gene expression, can contribute to several human disorders, but little is still known about the factors that regulate *MTHFR* methylation levels. We performed the present study to investigate if common polymorphisms in one-carbon metabolism genes contribute to *MTHFR* methylation levels. *MTHFR* methylation was assessed in peripheral blood DNA samples from 206 healthy subjects with methylation-sensitive high-resolution melting (MS-HRM); genotyping was performed for *MTHFR* 677C>T (rs1801133) and 1298A>C (rs1801131), *MTRR* 66A>G (rs1801394), *MTR* 2756A>G (rs1805087), *SLC19A1* (*RFC1*) 80G>A (rs1051266), *TYMS* 28-bp tandem repeats (rs34743033) and 1494 6-bp ins/del (rs34489327), *DNMT3A* -448A>G (rs1550117), and *DNMT3B* -149C>T (rs2424913) polymorphisms. We observed a statistically significant effect of the *DNMT3B* -149C>T polymorphism on mean *MTHFR* methylation levels, and particularly CT and TT carriers showed increased methylation levels than CC carriers. The present study revealed an association between a functional polymorphism of *DNMT3B* and *MTHFR* methylation levels that could be of relevance in those disorders, such as inborn defects, metabolic disorders and cancer, that have been linked to impaired DNA methylation.

## 1. Introduction

**Figure 1:**
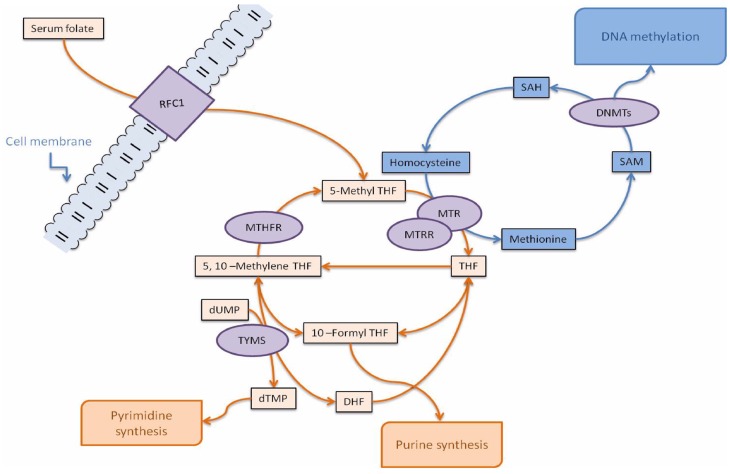
Simplified overview of the folate (orange color) and methionine (blue color) cycles in the one-carbon metabolism, adapted from [1]. The diagram illustrates the enzymes (violet color) whose polymorphisms have been investigated in this article, and their metabolites. Enzymes: DNMTs, DNA methyltransferases; MTHFR, methylenetetrahydrofolate reductase; MTR, methionine synthase; MTRR, methionine synthase reductase; RFC1, reduced folate carrier 1; TYMS, thymidilate synthase. Metabolites: DHF, dihydrofolate; THF, tetrahydrofolate; dTMP, deoxythymidine monophosphate; dUMP, deoxyuridine monophosphate; SAH, *S*-adenosylhomocysteine; SAM, *S*-adenosylmethionine.

The folate and methionine cycles are the “core” part of the one-carbon metabolism, a set of interconnected pathways that supply methyl groups for the synthesis of nucleic acids, amino acids, and *S*-adenosylmethionine (SAM), the main intracellular methylating agent [1]. Methylenetetrahydrofolate reductase (MTHFR) is a pivotal enzyme in one-carbon metabolism and catalyzes the conversion of 5,10-methylenetetrahydrofolate to 5-methyltetrahydrofolate (5-methylTHF), the main form of circulating folate and the methyl donor for homocysteine (hcy) remethylation to methionine in the reaction catalyzed by methionine synthase (MTR) that transfers the methyl group from 5-methylTHF to hcy, forming tetrahydrofolate (THF) and methionine. Methionine is then used for the production of SAM, required for DNA and protein methylation reactions, and THF re-enters the folate pathway as an acceptor of novel one-carbon moieties (Figure 1).

Rare and severe *MTHFR* mutations lead to MTHFR deficiency, an autosomal recessive inborn defect characterized by extremely high hcy levels in the urine and plasma, causing developmental delay, eye disorders, thrombosis, and osteoporosis [2]. More common polymorphisms in the *MTHFR* gene, such as the 677C>T (rs1801133) and 1298A>C (rs1801131) ones, reduce the enzyme thermostability and activity and can lead to hyperhomocysteinemia, especially in homozygous 677TT carriers [3,4,5]. These common polymorphisms, and particularly the *MTHFR* 677C>T one, have been often associated with a small increase in the risk of various human conditions, including male infertility, pregnancy loss, neural tube defects, congenital heart disease and Down syndrome [6,7,8], and have been suggested to contribute to complex disorders such as cancer, cardiovascular diseases, autoimmune disorders and neurodegenerative diseases, among others [9,10,11,12,13,14].

More recent evidence suggests than not only sequence variants, but also epigenetic modifications of the *MTHFR* gene can contribute to human disorders. Particularly, increased *MTHFR* promoter methylation results in decreased gene expression levels and has been associated with male infertility, pre-eclampsia, recurrent miscarriages, trisomy 21 and congenital heart disease in the offspring [15,16,17,18,19,20,21]. *MTHFR* hyper-methylation is also suspected to play a role in diabetic complications, vascular diseases and cancer [22,23,24,25,26].

Increasing evidence suggests an association between circulating folate levels and the methylation status of several genes [26,27,28], and common polymorphisms of genes involved in one-carbon metabolism, including *MTHFR* 677C>T and 1298A>C, *MTR* 2756A>G (rs1805087), methionine synthase reductase (*MTRR*) 66A>G (rs1801394), thymidilate synthase (*TYMS*) 28-bp tandem repeat (rs34743033) and 1494 ins/del (rs34489327), reduced folate carrier (*SLC19A1* or *RFC1*) 80G>A (rs1051266), and DNA methyltransferases *DNMT3A* -448A>G (rs1550117) and *DNMT3B* -149C>T (rs2424913) ones, have been frequently investigated as potential modulators of either global or gene-specific methylation levels in various human conditions [29,30,31,32]. In addition, there is evidence that certain polymorphisms, such as *MTR* polymorphisms, can act as cis-regulatory elements to regulate the methylation levels of their own gene promoter, as well as trans-regulatory elements to regulate the methylation levels of other metabolic genes [33]. However, little is still known concerning the contribution of common polymorphisms in one-carbon metabolism genes to *MTHFR* methylation levels.

To further address this issue, in the present study we investigated a cohort of 206 healthy individuals searching for correlation between common polymorphisms in the main genes of one-carbon metabolism and the methylation levels of the *MTHFR* gene.

## 2. Results

Table 1 shows the demographic characteristics of the study population and the average methylation levels of the *MTHFR* gene in our cohort. The study was performed in 206 healthy Italian subjects, including 67 males and 137 females of mean age 71.4 ± 15.4 years. We investigated a CpG island located in the 5′ untranslated region (5′ UTR) of the *MTHFR* gene, whose methylation levels are inversely correlated with gene expression levels [24,34]. Methylation levels of this region ranged from 5.9% to 69.3% in the study population, with an average value of 29.3%, which is in agreement with previous investigations in various populations [20,24,27,35].

Table 2 shows the genotype distribution of the investigated polymorphisms in the study population; all the genotype distributions conformed to Hardy-Weinberg equilibrium (HWE) expectations and are in agreement with those previously reported in healthy Caucasians [5,36,37].

**Figure 2:**
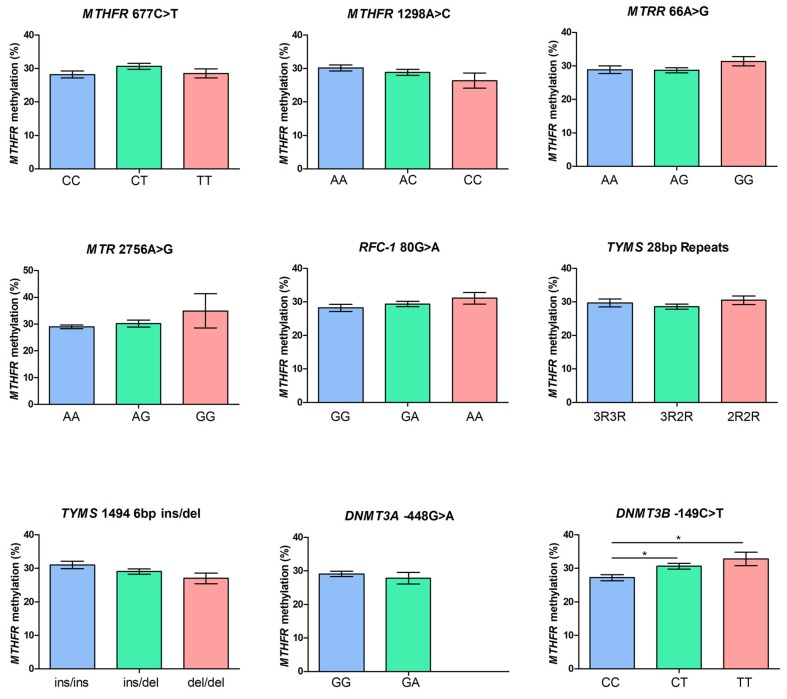
Correlation between one-carbon metabolism gene polymorphisms and *MTHFR* methylation levels. Data are expressed as means ± SEM. *Denotes a statistically significant difference after post-hoc Bonferroni’s correction for multiple testing (*p* < 0.05).

The correlation between each of the studied polymorphisms and *MTHFR* methylation levels is shown in Figure 2. We observed a statistically significant effect of the *DNMT3B* -149C>T polymorphism on mean *MTHFR* methylation levels, and in particular, a significant difference between wild-type (CC) and heterozygous (CT) subjects (27.2 ± 0.9% vs. 30.6 ± 0.9%, *p* = 0.02), and a significant difference between wild-type (CC) and mutant (TT) subjects (27.2 ± 0.9% vs. 32.8 ± 2.0%, *p* = 0.03), revealing that *MTHFR* methylation increases significantly with the increasing number of T alleles. None of the other polymorphisms showed a significant contribution to *MTHFR* methylation levels (Figure 2).

## 3. Discussion

Increasing evidence suggests that *MTHFR* hypermethylation represents a risk factor for various human disorders [15,16,17,18,19,20,21,22,23,24,25,26], but little is still known concerning the genetic factors acting as regulatory elements of *MTHFR* methylation levels. In the present study we investigated several of the major polymorphisms in one-carbon metabolism genes as potential modulators of *MTHFR* gene methylation in blood DNA samples from 206 healthy individuals, observing a statistically significant contribution of the *DNMT3B* -149C>T one. This polymorphism is located 149 base pairs upstream the transcription start site, and the minor T allele has been associated with increased *DNMT3B* gene expression levels compared to the major C one [38,39]. DNMT3B is the major *de novo* DNA methyltransferase expressed and active during the early stages of embryonic development, and is impaired in human diseases with chromosomal and genomic instabilities, including inherited diseases and cancer [40]. Particularly, in vitro studies revealed a 30% increased promoter activity of the *DNMT3B* -149T allele with respect to the C one [38]. Similarly, the *DNMT3B* -149T allele resulted in increased gene expression levels in human pancreatic cancer cells [39]. Therefore, it was suggested that the *DNMT3B* -149C>T polymorphism increases human cancer risk by increasing *DNMT3B* gene expression levels resulting in increased promoter methylation and silencing of tumor suppressor genes [38,39]. Indeed, this polymorphism was associated with increased risk of several cancers [40], including lung cancer in smokers [41], prostate and gastric and colorectal cancer in certain populations, albeit with conflicting results [42,43]. Furthermore, it was shown that the *DNMT3B* -149C>T polymorphism was linked to altered methylation levels of cancer related genes, such as h*MLH1* and *ECAD*, in colorectal cancer cells [44].

Folate metabolism is required in all dividing cells for a proper supply of nucleotides, as well as in non-dividing cells such as neurons, for a proper repair of damaged DNA bases and for the regulation of DNA and protein methylation patterns, and therefore most of the folate-related genes are ubiquitously expressed and differentially regulated in human tissues, including blood cells [1]. Particularly, there is an indication that both *DNMT3A* and *DNMT3B* are de-methylated and expressed blood cells of healthy individuals [35,45]. Similarly, the *MTHFR* is expressed in blood cells [46], and the expression levels are inversely regulated by promoter methylation levels, that show a large inter-individual variability [24,35].

Indeed, the contribution of *DNMT3B* polymorphisms to human disease has been investigated in many other diseases than cancer. For example, increasing evidence suggests that the *DNMT3B* -149C>T polymorphism, either alone or in haplotype combination with other non-coding *DNMT3B* polymorphisms, contributes to the maternal risk for having a child with Down syndrome [47,48,49], and has been associated with an increased risk of prematurity [30], with childhood immune thrombocytopenia [50,51] and autoimmune thyroid disease [52]. The association of the *DNMT3B* -149C>T polymorphism with neurological and neurodegenerative diseases, either alone or in haplotype combination, is still controversial [53,54,55,56].

Present findings linking the *DNMT3B* -149C>T polymorphism to *MTHFR* methylation levels are original, and if confirmed in other populations could be of relevance for those conditions characterized by increased *MTHFR* methylation, and particularly for congenital disorders or cancers associated with *DNMT3B* polymorphisms. For example, recent studies revealed increased *MTHFR* methylation as a risk factor for recurrent miscarriages, as well as for Down syndrome and congenital heart disease in the offspring [18,20,21], and *MTHFR* hyper-methylation was seen in several human cancers [24,25,34,57]. Also, male infertility has been recently linked to a *DNMT3B* polymorphism in strong linkage with the -149C>T one [58], and several studies suggest association of *MTHFR* hyper-methylation with male infertility [15,16,17]. Therefore, a further investigation of the link between *DNMT3B* polymorphisms and *MTHFR* methylation levels is warranted in several human disorders.

DNMT3A is the other *de novo* DNA methyltransferase, and the *DNMT3A* -448A>G polymorphisms was recently associated with the risk of spontaneous abortion [59], as well as with risk of several cancers [60]; therefore, we decided to investigate its contribution to *MTHFR* methylation levels, observing no association. To the best of our knowledge, there are no other studies addressing this issue.

Concerning *MTHFR* 677C>T and 1298A>C polymorphisms, we found no association with *MTHFR* methylation levels. A previous study performed in 101 epileptic patients treated with valproic acid (VPA) and 68 healthy controls also reported no association of *MTHFR* 677C>T and 1298A>C polymorphisms with *MTHFR* gene methylation levels in blood DNA of both groups [61]. Collectively, present and previous data [61] suggest that *MTHFR* 677C>T and 1298A>C polymorphisms are unlikely to act as cis-regulatory modulators of *MTHFR* gene methylation levels. In that manuscript the authors also investigated the contribution of *MTR* 2756A>G, *MTRR* 66A>G, and *RFC-1* 80G>A to *MTHFR* methylation levels, observing an association of the *MTR* 2756A>G polymorphism only in epileptic patients treated with VPA, but none of the three polymorphisms was linked to *MTHFR* methylation levels in healthy controls [61]. In the present investigation we included only healthy individuals not taking drugs or supplements known to affect the epigenome, and present data are in agreement with that previous investigation [61], indicating that *MTR* 2756A>G, *MTRR* 66A>G, and *RFC-1* 80G>A polymorphisms are not associated with *MTHFR* methylation levels in untreated healthy subjects. VPA is a very potent epigenetic drug, exerting anti-cancer and neuro-protective effects by its inhibitory action on proteins that catalyze histone deacetylation [60]. Several recent studies suggest that VPA also induces changes is serum folate and hcy levels, as well changes in the methylation levels of several genes, including *MTHFR* [59,60,61]. Therefore, the contribution of polymorphisms in one-carbon metabolism genes to global and gene-specific methylation levels might be exacerbated under treatments that globally affect folate metabolism and the epigenome [61,62,63].

TYMS competes with MTHFR for 5,10-methylenetetrahydrofolate, and the folate pathway can be shifted toward the synthesis of DNA precursors or toward hcy remethylation to methionine, depending on the cell requirements (Figure 1). We previously observed association of *TYMS* polymorphisms with the methylation levels of tumor suppressor and DNA repair genes in colorectal cancer cells [31], but no previous study investigated the contribution of *TYMS* polymorphisms to *MTHFR* gene methylation. However, the present investigation revealed no association of *TYMS* polymorphisms with *MTHFR* methylation levels.

In summary, we investigated the contribution of several of the major polymorphisms of genes coding enzymes involved in the folate and methionine cycles of the one-carbon metabolism to *MTHFR* methylation levels in healthy individuals, observing a significant association of the *DNMT* -149T allele with increased *MTHFR* methylation. Additional studies are required to further address this issue in human disorders characterized by *MTHFR* hyper-methylation, as well as to investigate if drugs or compounds exerting epigenetic properties are able to modulate this association. Of particular interest are disorders such as Down syndrome, lung and gastrointestinal cancers, and male infertility, all linked to *DNMT3B* variants and characterized by *MTHFR* hyper-methylation.

## 4. Materials and Methods

### 4.1. Study Population

DNA samples from peripheral leukocytes were available from a total of 206 healthy individuals, including 67 males and 139 females of mean age 71.4 ± 15.4 years (Table 1), mainly recruited from 2011 to 2015 as healthy control subjects for genetic and epigenetic case-control investigations [13,20,35]. All the individuals were volunteer subjects of Italian origin, underwent a rigorous clinical and neurological examination, and were healthy at blood drawing. In addition, individuals taking vitamins, drugs, substances or supplements known or suspected to interfere with one-carbon metabolism and DNA methylation reactions, such as anti-cancer drugs, anti-epileptic drugs, anti-inflammatory drugs, epi-drugs, metformin, tobacco smoking, folic acid or other vitamin supplements, were not enrolled in the study. Each subject gave an informed and written consent for the inclusion in the study that received approval from the Ethics Committee of the Pisa University Hospital (Protocol number 3618/2012), and was performed in accordance with the Declaration of Helsinki.

### 4.2. Analysis of MTHFR Methylation Levels

Two hundred nanograms of DNA from each sample have been treated with sodium bisulfite in order to convert un-methylated cytosines into uracil, using the EpiTect Bisulfite Kit (Qiagen, Milan, Italy, Catalog N° 59104). Bisulfite conversion was performed simultaneously on all samples in order to avoid potential batch effects, and the bisulfite conversion efficiency was assessed using a sample of completely un-methylated human DNA (Qiagen, Catalog N° 59568), resulting of 99% in average. The *MTHFR* methylation levels have been assessed using a methylation-sensitive high-resolution melting (MS-HRM) protocol previously developed in our laboratory, validated by pyrosequencing, and fully described by us elsewhere [32]. All the MS-HRM analyses were performed using a CFX96 Real-Time PCR detection system (Bio-Rad, Milan, Italy). Particularly, we studied a CpG island in the 5′-untranslated (UTR) region of the *MTHFR* gene spanning from +30 to +184 from the transcription start site, and containing 7 CpG sites whose methylation levels were found to be inversely correlated with *MTHFR* gene expression levels by several authors [24,34]. Table 3 shows the sequence of the primers, the annealing temperature (*T*_a_), the studied region, the length of the amplicon, and the number of CpG sites within it. Each reaction was performed in duplicate, and we analyzed 10% of the samples independently on separate occasions to verify the inter-assay variability. Fully methylated and un-methylated DNA (EpiTectH methylated and unmethylated human control DNA, bisulfite converted, Qiagen, Catalog N° 59695) were mixed to obtain the following ratios of methylation: 0%, 12,5%, 25%, 50%, 75%, 100%. Standard DNA samples with known methylation ratios were included in each assay in order to generate standard curves that were used to deduce the methylation levels of each sample, using an interpolation method previously described [64].

### 4.3. Analysis of Common Polymorphisms in One-Carbon Metabolism Genes

Genotyping for *MTHFR* 677C>T (rs1801133), *MTHFR* 1298A>C (rs1801131), *MTRR* 66A>G (rs1801394), *MTR* 2756A>G (rs1805087), *SLC19A1* (*RFC-1*) 80G>A (rs1051266), *TYMS* 28-bp repeats (rs34743033), *TYMS* 1494 6-bp ins/del (rs34489327), *DNMT3A* -448A>G (rs1550117), and *DNMT3B* -149C>T (rs2424913) polymorphisms was performed by PCR/RFLP techniques as detailed elsewhere [36,65].

### 4.4. Statistical Analysis

To verify that genotype frequencies of all the studied polymorphisms were in Hardy-Weinberg equilibrium we used the Chi-square (Χ^2^) analysis. *MTHFR* methylation data were tested for normality using the Shapiro-Wilk test and showed a normal distribution in our cohort. Multi-factor analysis of variance (ANOVA) including age at sampling, gender, and all the other investigated polymorphisms as covariates, was used to investigate the contribution of each studied polymorphism to *MTHFR* methylation levels, followed by post-hoc Bonferroni’s correction for multiple testing. Statistical analyses were performed with the STATGRAPHICS 5.1 plus software package for Windows, and Bonferroni’s corrected *p*-values < 0.05 were considered statistically significant.

## Figures and Tables

**Table 1:** Study population.

Total Subjects	Age (Mean ± SD)	Gender	*MTHFR* Methylation (Mean ± SD)
206	71.4 ± 15.4	M: 67	29.3 ± 9.3%
		F: 139	

**Table 2:** Distribution of genotypes in the study population.

Polymorphism	Genotypes: N° of Subjects (%)
*MTHFR* 677C>T	CC: 72 (35.0%), CT: 91 (44.2%), TT: 43 (20.8%)
*MTHFR* 1298A>C	AA: 95 (46.1%), AC: 95 (46.1%), CC: 16 (7.8%)
*MTRR* 66A>G	AA: 61 (29.6%), AG: 105 (51.0%), GG: 40 (19.4%)
*MTR* 2756A>G	AA: 157 (76.2%), AG: 47 (22.8%), GG: 2 (1.0%)
*RFC-1* 80G>A	GG: 62 (30%), GA: 113 (54.9%), AA: 31 (15.1%)
*TYMS* 28bp Repeats	3R3R: 52 (25.2%), 3R2R: 108 (52.4%), 2R2R: 46 (22.4%)
*TYMS* 1494 6bp ins/del	ins/ins:64 (31.1%), ins/del: 108 (52.4%), del/del: 34 (16.5%)
*DNMT3A* -448G>A	GG: 170 (82.3%), GA: 36 (17.7%), AA: 0 (0.0%)
*DNMT3B* -149C>T	CC: 90 (43.7%), CT: 96 (46.6%), TT: 20 (9.7%)

**Table 3:** Sequence of the primers, annealing temperature (*T*_a_), length of the amplicon, studied region and number of CpG sites.

Primer Sequences	*T*_a_	Amplicon Lenght	Region	CpG Sites
F: 5′-TTTTAATTTTTGTTTGGAGGGTAGT-3′ R: 5′-AAAAAAACCACTTATCACCAAATTC-3′	54 °C	155 bp	From +30 to +184	7

## Abbreviations

DNMT	DNA methyltransferase
HWE	Hardy-Weinberg equilibrium
MS-HRM	Methylation-sensitive high-resolution melting
MTHFR	Methylenetetrahydrofolate reductase
MTR	Methionine synthase
MTRR	Methionine synthase reductase
RFC1	Reduced folate carrier 1
SAM	*S*-adenosylmethionine
SD	Standard deviation
SEM	Mean standard error
THF	Tetrahydrofolate
TYMS	Thymidilate synthase
VPA	Valproic acid
